# Preprocessed Consortium for Neuropsychiatric Phenomics dataset

**DOI:** 10.12688/f1000research.11964.2

**Published:** 2017-09-22

**Authors:** Krzysztof J. Gorgolewski, Joke Durnez, Russell A. Poldrack

**Affiliations:** 1 Department of Psychology, Stanford University, Stanford, CA, USA; 2 INRIA Parietal, Neurospin, Saclay, Gif-sur-Yvette, France

**Keywords:** fMRI, human, cognition, preprocessed

## Abstract

Here we present preprocessed MRI data of 265 participants from the Consortium for Neuropsychiatric Phenomics (CNP) dataset. The preprocessed dataset includes minimally preprocessed data in the native, MNI and surface spaces accompanied with potential confound regressors, tissue probability masks, brain masks and transformations. In addition the preprocessed dataset includes unthresholded group level and single subject statistical maps from all tasks included in the original dataset. We hope that availability of this dataset will greatly accelerate research.

## Introduction

Recently, the Consortium for Neuropsychiatric Phenomics published a dataset ^1^ with neuroimaging as well as phenotypic information for 272 participants. The subject population consists of healthy controls (130 subjects), as well as participants with diagnoses of adult ADHD (43 subjects), bipolar disorder (49 subjects) and schizophrenia (50 subjects). The goal of the study is to examine brain function and anatomy for these common neuropsychiatric syndromes. The study focuses on memory and response inhibition, with a large battery of questionnaires, neurocognitive tasks, a neuropsychological assessment and multiple neuroimaging modalities. Details on the complete assessment for each subject can be found in the data descriptor ^1^. It is undoubtedly a rich resource for the academic community that can help shed light on the relationship between brain and behavior, especially with respect to neuropsychiatric disorders. However, before any brain-behaviour relationships can be answered, computationally expensive processing steps need to be performed ^2^. In addition to requiring a substantial amount of computing resources, a certain level of expertise in MRI data processing and fMRI task modelling is required before the data can be used to test scientific hypotheses.

To facilitate answering scientific questions using the CNP dataset, we have performed standard preprocessing as well as statistical modeling on the data, and are making the results of these analyses openly available. The preprocessing was designed to facilitate a wide range of analyses, and includes outputs in native (aligned with participants T1 weighted scan), MNI (volumetric) and fsaverage5 (surface) spaces. The data have not been denoised, but potential confound regressors have been calculated for each run, giving researchers the freedom to choose their own denoising schemes. In addition, we also include group and single subject statistical maps for all tasks available in the original dataset. This preprocessed dataset joins the ranks of similar initiatives for other openly shared datasets ^3– 5^, and we hope it will be equally useful to the scientific community.

The processed data can be found alongside the original unprocessed data in the OpenfMRI repository ^6^ under the revision 1.0.4.

## Methods

### Participants and procedures

The sample of subjects contains 155 men and 117 women, with ages between 21 and 50 years (mean: 33.23; median: 31.0). Each subject completed at least 8 years of formal education and have either English or Spanish as primary language. Subjects were recruited by community advertisement and through outreach to local clinics and online portals. The consortium excluded patients with diagnoses in at least 2 different patient groups. Furthermore, the following exclusion criteria were used: left-handedness, pregnancy, history of head injury with loss of consciousness or other contraindications to scanning.

Neuroimaging data were acquired on a 3T Siemens Trio scanner. Functional MRI data were collected with a T2*-weighted echoplanar imaging (EPI) sequence with parameters: slice thickness = 4mm, 34 slices, TR=2s, TE=30ms, flip angle=90°, matrix=64 × 64, FOV=192mm. A T1-weighted high-resolution anatomical scan (MPRAGE) were collected with the following parameter: slice thickness = 1mm, 176 slices, TR=1.9s, TE=2.26ms, matrix=256 x 256, FOV=250mm. Diffusion weighted imaging data were collected with parameters: slice thickness = 2mm, 64 directions, TR/TE=9000/93ms, flip angle=90°, matrix=96 × 96, axial slices, b=1000s/mm ^2^.

The following fMRI protocols were used (see full details in 1):

a. A resting state fMRI session of 304 seconds (eyes open)

b. *Balloon analog risk task.* Participants were allowed to pump a series of virtual balloons. Experimental balloons (green) resulted either in an explosion or in a successful pump (no explosion and 5 points). Control (white) balloons did not result in points nor exploded. Participants could choose not to pump but to cash out and start with a new balloon.

c. *Paired associate memory task* including a memory encoding task and a retrieval task. During the initial memory encoding task, two words were shown. Line drawings of those two objects were added after 1 second. During control trials, the line drawings were replaced with scrambled stimuli. On each trial, one of the drawings was black and white, while the other object was colored. Subjects were instructed to indicate by button press the side of the colored object. During the retrieval task, subjects were shown a pair of objects and rate their confidence in their memory of the pairing with response options ranging from Sure correct to Sure incorrect. During control trials, one of the response options was shown on side of the screen and ‘XXXX’ on the other side of the screen. Subjects were asked to press the button that corresponded to the response option displayed.

d. *Spatial working memory task*. Subjects were shown an array of 1, 3, 5 or 7 circles pseudorandomly positioned around a central fixation cross. After a delay, subjects were shown a green circle and were asked to indicate whether the circle was in the same position as one of the target circled. In addition to the memory load, the delay period was manipulated with delays of 1.5, 3 or 4.5s. Half the trials were true-positive and half were true negative.

e. Stop signal task. Participants were instructed to respond quickly when a ‘go’ stimulus was presented on the computer screen, except on the subset of trials where the ‘go’ stimulus was paired with a ‘stop’ signal. The ‘go’ stimulus was a pointing arrow, a stop-signal was a 500 Hz tone presented through headphones.

f. *Task-switching task.* Stimuli were shown varying in color (red or green) and in shape (triangle or shape). Participants were asked to respond to the stimulus based on the task cue (shape ‘S’ or color ‘C’). The task switched on 33% of the trials.

g. *Breath holding task.* Participants were asked to alternate between holding their breath and breathing regularly while resting.

The procedures were approved by the Institutional Review Boards at UCLA and the Los Angeles County Department of Mental Health.

### Data processing overview

**Figure 1.**
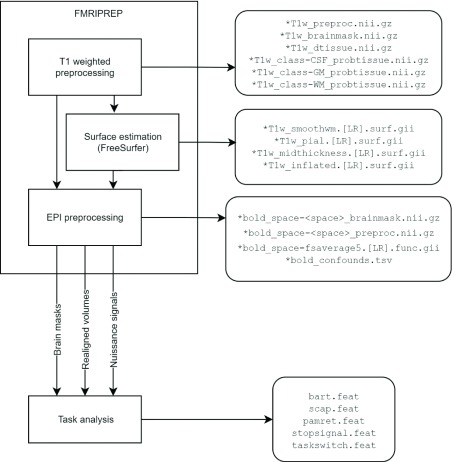
Overview of data processing and selected outputs.

Data processing has been split into preprocessing and task analysis (model fitting). For an overview see Figure 1.

### Preprocessing

The input dataset was acquired from OpenfMRI.org ^6^ - accession number ds000030, revision 1.0.3. Even though the original dataset included data from 272 participants, seven were missing T1 weighted scans (see Table 1) and thus only data from 265 participants were preprocessed.

**Table 1.** Known issues. List of problems with the raw data we were aware of at the time of writing that impacted preprocessing.

Participants affected	Issue
10971, 10501, 70036, 70035, 11121, 10299, 10428	Lack of T1w files. Preprocessing and task modelling was not performed.
11067	Signal dropout in the cerebellum during BART, rest, SCAP, stop-signal and task switch tasks.

Results included in this manuscript come from preprocessing performed using FMRIPREP version 0.4.4 ( http://fmriprep.readthedocs.io). This recently developed tool is a robust preprocessing pipeline based on the Nipype workflow engine ^7^. FMRIPREP aims at combining different implementations of various MR signal processing algorithms (from established software packages such as FSL, AFNI, or ANTs) to deliver a robust spatial normalization and nuisance estimation workflow. The tool was run with the following command line arguments:

--participant_label {sid} -w $LOCAL_SCRATCH --output-space T1w fsaverage5 template --nthreads 8 --mem_mb 20000

Where { sid} was the participant label and $LOCAL_SCRATCH was temporary folder for storing intermediate results.

Within the pipeline each T1 weighted volume was corrected for bias field using ANTs N4BiasFieldCorrection v2.1.0 ^8^, skullstripped using antsBrainExtraction.sh v2.1.0 (using OASIS template), and coregistered to skullstripped ICBM 152 Nonlinear Asymmetrical template version 2009c ^9^ using symmetric image normalization method (SyN) nonlinear transformation with affine initialization implemented in ANTs v2.1.0 ^10^.

Cortical surface was estimated from the bias field corrected T1 weighted volume (in subject space) using FreeSurfer v6.0.0 ^11^. Due to its high quality, the brain mask derived by antsBrainExtraction.sh was used in the FreeSurfer pipeline instead of relying on the skullstripping algorithm included in FreeSurfer.

Functional data for each run was motion corrected using MCFLIRT v5.0.9 ^12^. Functional data was skullstripped using combination of BET (from FSL) and 3dAutoMask (from AFNI) tools and was coregistered to the corresponding T1 weighted volume using boundary based registration with 9 degrees of freedom - implemented in FreeSurfer v6.0.0 ^13^. Motion correcting transformations, transformation to T1 weighted space and MNI template warp were applied in a single step using antsApplyTransformations v2.1.0 with Lanczos interpolation.

Three tissue classes were extracted from T1 weighted images using FSL FAST v5.0.9 ^14^. Voxels from cerebrospinal fluid and white matter were used to create a mask in turn used to extract physiological noise regressors using the principal component analysis-based method known as aCompCor ^15^. The mask was eroded and limited to subcortical regions to limit overlap with grey matter, six principal components were estimated. Framewise displacement and dvars ^16^ was calculated for each functional run using Nipype implementation. In addition to those regressors global signal and mean white matter signal was also calculated.

The whole dataset was preprocessed on the Stanford Sherlock supercomputer in total three times. After each iteration the decision to modify the preprocessing was purely based on the visual evaluation of the preprocessed data and not based on results of model fitting. First iteration (using FMRIPREP 0.4.2) uncovered inconsistent output image field of view and issues with EPI skullstripping, second iteration (using FMRIPREP 0.4.3) uncovered two cases of failed normalization due to poor initialization. In the final iteration all those issues were resolved. In total, the preprocessing consumed ~22,556 single CPU hours.

For more details of the pipeline see http://fmriprep.readthedocs.io/en/0.4.4/workflows.html (also archived in the Internet Archive at https://web.archive.org/web/20170913233706/http://fmriprep.readthedocs.io/en/0.4.4/workflows.html).

### Volume-based task analysis

For a full description of the paradigms for each task, please refer to 1. We analysed the task data using FSL ^17^ and AFNI ^18^, implemented using Nipype ^7^. Spatial smoothing was applied using AFNI’s 3dBlurInMask with a Gaussian kernel with FWHM=5mm. Activity was estimated using a general linear model (GLM) with FEAT ^17^. Predictors were convolved with a double-gamma canonical haemodynamic response function ^19^. Temporal derivatives were added to all task regressors to compensate for variability in the haemodynamic response function. Furthermore, the following regressors were added to avoid confounding due to motion: standardised dvars, absolute dvars, the voxelwise standard deviation of dvars, framewise displacement, and the six motion parameters (translation in 3 directions, rotation in 3 directions).

For the Balloon Analog Risk Task (BART), we included 9 task regressors: for each condition (accept, explode, reject), we added a regressor with equal amplitude and durations of 1 second on each trial. Furthermore, we included the same regressors with the amplitude modulated by the number of trials before explosions (perceived as the probability of explosions). The modulator was mean centered to avoid estimation problems due to collinearity. For the conditions that require a response (accept, reject), a regressor was added with equal amplitude, and the duration equal to the reaction time. These regressors were orthogonalised with their fixed-duration counterpart to separate the fixed effect of the trial and the effect covarying with the reaction time. A regressor is added for the control condition.

In the retrieval phase of the Paired-Associate Memory Task (PAMRET), we modelled 4 conditions: true positives, false positives, true negatives, false negatives. For each condition, a regressor is modelled first with fixed durations (3s) and second with reaction time durations, with the latter orthogonalised with the former. With an extra regressor with control trials, there are 9 task regressors in total.

In the Spatial Capacity Task (SCAP), 25 task regressors were included. For each cognitive load (1 - 3 - 5 - 7) and each delay (1.5 - 3 - 4.5) with a correct response, two regressors were added: a regressor with fixed durations of 5 seconds and one with the duration equal to the reaction time, with the second orthogonalised with respect to the first. For both regressors, the onset is after the delay. The last regressor summarises all incorrect trials.

For the Stop-Signal Task (STOPSIGNAL), for each condition (go, stop - successful, stop - unsuccessful), one task regressor was included with a fixed duration of 1.5s. For the conditions requiring a response (go and stop-unsuccessful), an extra regressor was added with equal amplitude, but the duration equal to the reaction time. Again, these regressors were orthogonalised with respect to the fixed duration regressor of the same condition. A sixth regressor was added with erroneous trials.

In the Task Switching Task (TASKSWITCH), all manipulations were crossed (switch/no switch, congruent/incongruent, CSI delay short/long), resulting in 8 task conditions. As in the SCAP task, we added for each condition two regressors: a regressor with fixed durations of 1 second, and one with the duration equal to the reaction time, with the second orthogonalised with respect to the first. There is a total of 16 regressors.

Not all subjects performed all tasks. Furthermore for subjects who are missing at least one regressor used in the contrasts, the task data are discarded. This is the case for example when no correct answers are registered for a certain condition in the SCAP task. For the SCAP task, we discarded 16 subjects; 14 subjects were removed for TASKSWITCH, 2 subjects for STOPSIGNAL, 2 subjects for BART, and 12 for PAMRET. Thus the total number of subjects modelled in the BART task is 259, while 244 subjects were modelled for the SCAP task. 254 subjects were included the TASKSWITCH task analysis, 197 subjects in the PAMRET task and 255 subjects in the STOPSIGNAL task.

All modelled contrasts are listed in the Supplementary material. As is shown, all contrasts are estimated and tested for both a positive and a negative effect.

### Group level analysis

Subsequent to the single subject analyses, all subjects were entered in a one-sample group level analysis for each task. Three second level analysis strategies were followed: (A) ordinary least squares (OLS) mixed modelling using FLAME ^17^, (B) generalized least squares (GLS) with a local estimate of random effects variance, using FSL ^17^, and (C) non-parametric modelling (NP) using RANDOMISE ^20^, with the whole brain first level parameter estimates for each subject as input, and 10,000 permutations. The first two analyses use a group brain mask with voxels that were present in 100% of all subjects, to ensure equal degrees of freedom in each voxel. For the permutation tests, a group mask was created where voxels were discarded for further analysis if less than 80% of the subjects have data in those voxels, to cover a larger part of the brain, especially in more remote area’s.

In addition to group level statistical maps, activation count maps (ACMs) were generated to show the proportion of participants that show activation, rather than average activation over subjects ^21^. These maps indicate whether the effects discovered in the group analyses are consistent over subjects. As in 21, the statistical map for each subject is binarized at z=+/-1.65. For each contrast, the average of these maps is computed over subjects. The average negative map (percentage of subjects showing a negative effect with z < -1.65) is subtracted from the average positive map to indicate the direction of effects.

## Selected results

**Figure 2.**
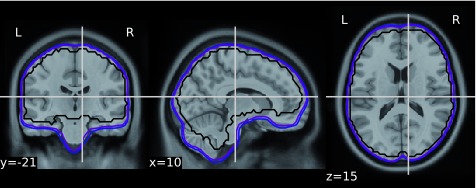
Overlap of the EPI derived 1,969 brain masks in the MNI space: voxels inside the blue outlined were present within the mask for 85% of runs, purple: 95% of runs, black 100% of runs. Animated visualizations of all coregistrations are available inside the HTML reports included as part of this dataset.

**Figure 3.**
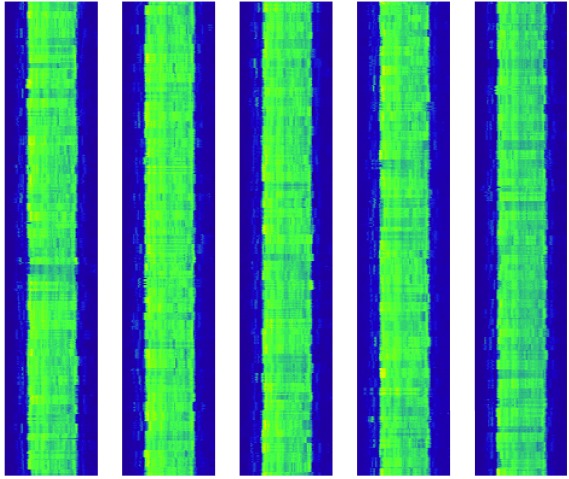
Visualization of the coregistration quality (hyperslab). Each line in all columns represents a single line of corresponding voxels from 1,969 preprocessed EPI images in MNI space (voxel coordinates i=20, k=50, t=10).

To validate the quality of volumetric spatial normalization we have looked at the overlap of the EPI derived brain masks in the MNI space (across all participants and runs - total of 1,969 masks - see Figure 2) and visualized alignment of a single line of voxels across all runs (see Figure 3). The within subject coregistration and between subject normalization worked well for the vast majority of participants, creating a very good overlap. All of the issues observed while processing the dataset are listed in Table 1.

**Figure 4.**
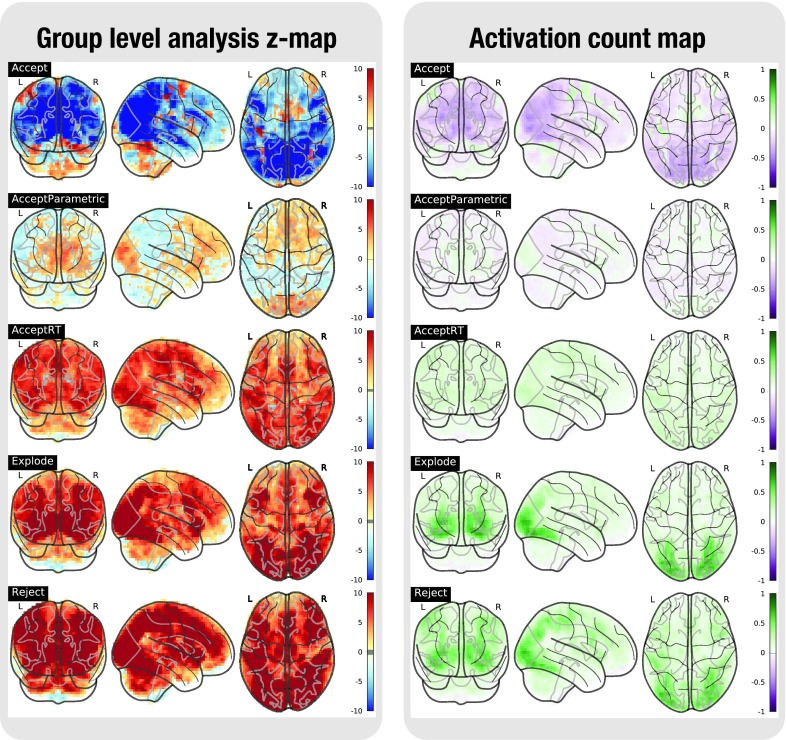
Task analysis results for the BART task. In the left plot, the statistical map of the one-sample group test, computed with randomise. The right plot shows the difference between the positive and the negative activation count maps.

**Figure 5.**
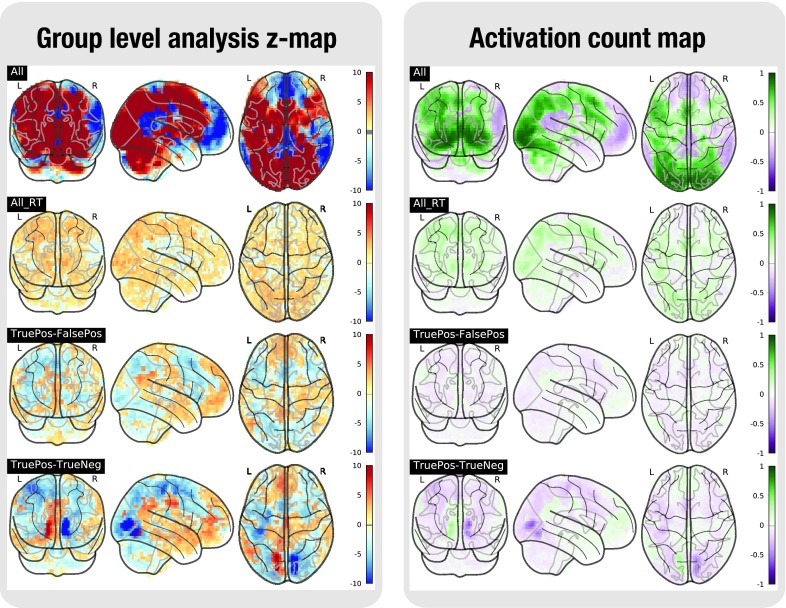
Task analysis results for the PAMRET task. In the left plot, the statistical map of the one-sample group test, computed with randomise. The right plot shows the difference between the positive and the negative activation count maps.

**Figure 6.**
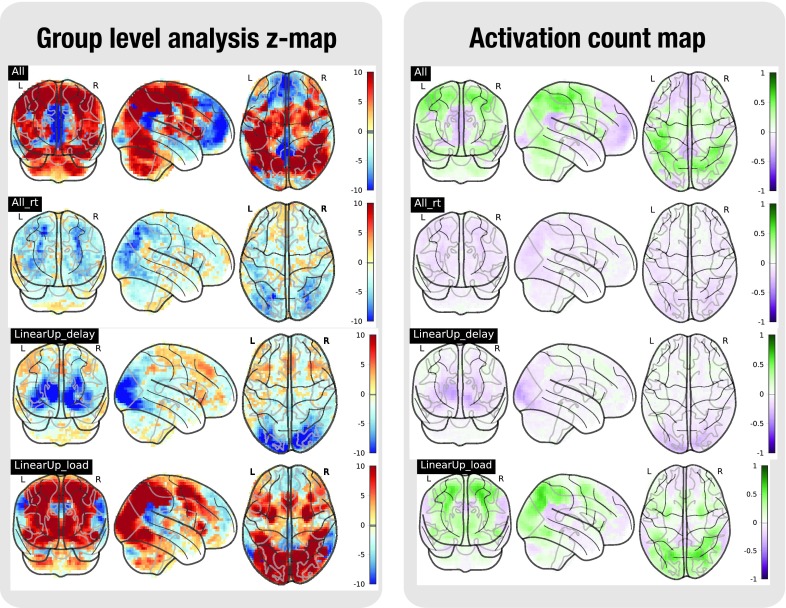
Task analysis results for the SCAP task. In the left plot, the statistical map of the one-sample group test, computed with randomise. The right plot shows the difference between the positive and the negative activation count maps.

**Figure 7.**
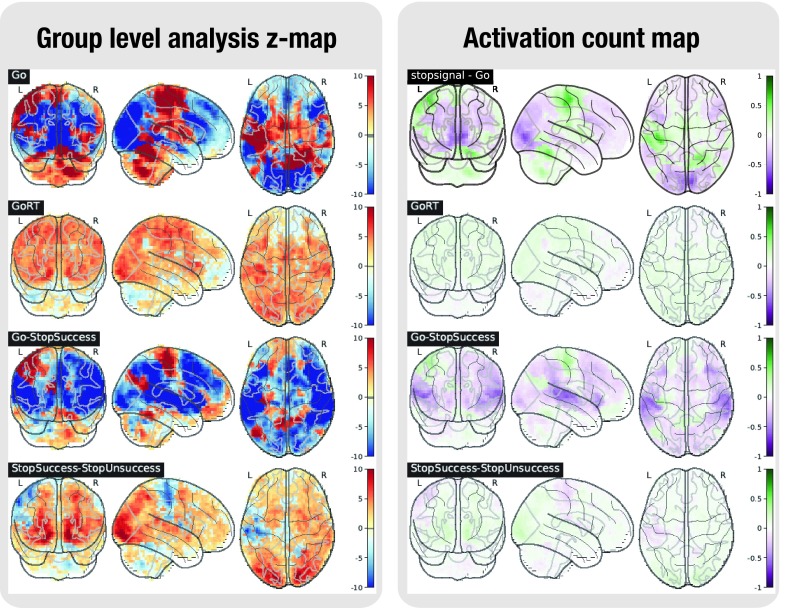
Task analysis results for the STOPSIGNAL task. In the left plot, the statistical map of the one-sample group test, computed with randomise. The right plot shows the difference between the positive and the negative activation count maps.

**Figure 8.**
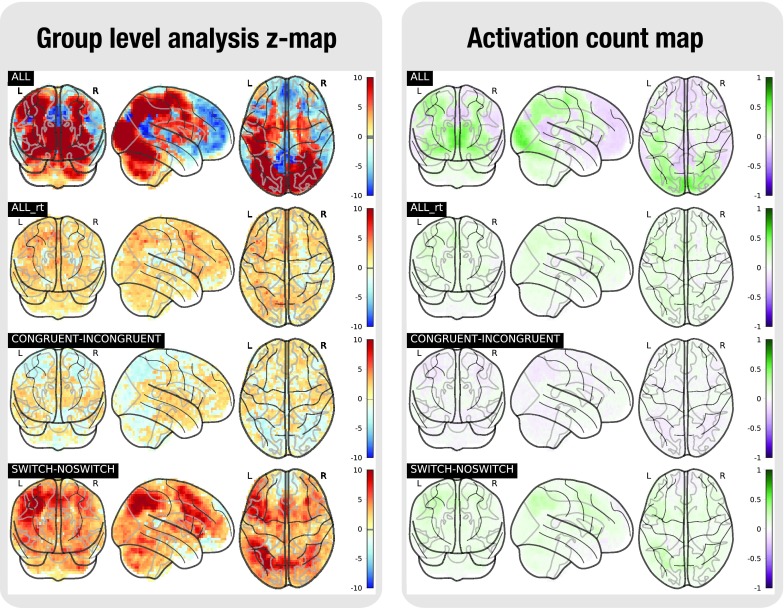
Task analysis results for the TASKSWITCH task. In the left plot, the statistical map of the one-sample group test, computed with randomise. The right plot shows the difference between the positive and the negative activation count maps.

A selection of the tested contrasts in the task analyses is shown in Figures 4 to 8. Figures were generated using nilearn ^22^.

## Data and software availability

The preprocessed images were deposited along the original dataset in the OpenfMRI repository – accession number: ds000030 ^6^, under the revision 1.0.4. The preprocessed data is organized according the draft extension to the Brain Imaging Data Structure (BIDS – see 23) specification for describing derived data. All FMRIPREP derivatives are organized under fmriprep/sub-<participant_label>/

Derivatives related to T1 weighted files are in the anat subfolder:

*T1w_preproc.nii.gz - bias field corrected T1 weighted file, using ANTS’ N4BiasFieldCorrection
* T1w_brainmask.nii.gz - brain mask derived using ANTS
*T1w_dtissue.nii.gz -tissue class map derived using FAST.
*T1w_class-CSF_probtissue.nii.gz, *T1w_class-GM_probtissue.nii.gz, *T1w_class-WM_probtissue.nii.gz - probability tissue maps.

All of the above are available in native and MNI space.

*T1w_smoothwm.[LR].surf.gii - smoothed gray white matter interface surfaces.
*T1w_pial.[LR].surf.gii - pial surface.
*T1w_midthickness.[LR].surf.gii - MidThickness surfaces.
*T1w_inflated.[LR].surf.gii - FreeSurfer inflated surfaces for visualization.
*T1w_space-MNI152NLin2009cAsym_class-CSF_probtissue.nii.gz, *T1w_space-MNI152NLin2009cAsym_class-GM_probtissue.nii.gz, *T1w_space-MNI152NLin2009cAsym_class-WM_probtissue.nii.gz - probability tissue maps, transformed into MNI space.
*T1w_target-MNI152NLin2009cAsym_warp.h5 Composite (warp and affine) transform to transform participant's T1 weighted image into the MNI space (HDF5 format).

Derivatives related to EPI files are in the func subfolder:

*bold_space-<space>_brainmask.nii.gz Brain mask for EPI files.
*bold_space-<space>_preproc.nii.gz Motion-corrected (using MCFLIRT for estimation and ANTs for interpolation) EPI file

All of the above are available in the native T1 weighted space as well as the MNI space.

*bold_space-fsaverage5.[LR].func.gii Motion-corrected EPI file sampled to surface.
*bold_confounds.tsv A tab-separated value file with one column per calculated confound (see Methods) and one row per timepoint/volume.

File formats: files with the .nii.gz extension are in the NIfTI file format (see https://nifti.nimh.nih.gov/), files with the .gii are in the GIfTI file format (see https://www.nitrc.org/projects/gifti/).

In addition, the dataset includes 265 visual quality HTML reports (one per participant) generated by FMRIPREP that illustrate all mayor preprocessing steps (T1 skullstripping, T1 to MNI coregistration, EPI skullstripping, EPI to T1 coregistration, and CompCor regions of interest).

All the FreeSurfer derivatives are organized under freesurfer/sub-<participant_label>/ according to the FreeSurfer native file organization scheme.

The results of the single subject task modeling are available in task/sub-<participant_label>/ and the group level results can be found in task_group/. Each subject-specific folder holds 5 folders - bart.feat, scap.feat, pamret.feat, stopsignal.feat, taskswitch.feat - with the results from the respective task modeling, organised as standard FEAT output. The group-level folder contains a folder for every task, in turn containing a folder for each contrast (see Supplementary material for naming conventions) and below those folders are the results of the three modeling strategies.

The results for each contrast in the one-sample group task analyses are deposited and can be interactively viewed in NeuroVault ^24^: http://neurovault.org/collections/2606/.

Latest source code used to produce the task analyses: https://github.com/poldracklab/CNP_task_analysis

Archived source code as at the time of publication: http://doi.org/10.5281/zenodo.832319 ^25^. License: MIT license.

All code has been run through a singularity container ^26^, created from a docker container poldracklab/cnp_task_analysis:1.0 available on Docker Hub ( https://hub.docker.com/r/poldracklab/cnp_task_analysis/).

To ensure long term preservation, the code has been shared on Zenodo and assigned a DOI. This does not only allow re-running of the analyses, but also regeneration of the singularity container with all necessary dependencies to do so. Furthermore, the data shared on NeuroVault and OpenfMRI are periodically archived in Stanford Digital Repository.

## Supplementary material

Supplementary File 1: Task fMRI Contrasts.
