# Supplementary material for: Preprocessed Consortium for Neuropsychiatric Phenomics dataset
Source: F1000Res. 2017 Sep 22;6:1262. Originally published 2017 Jul 28. [Version 2] doi: 10.12688/f1000research.11964.2 (PMC5664981; doi:10.12688/f1000research.11964.2)
Supplement: Supplementary file 1 [file f1000research-6-13763-s0000.tgz › 1b8f550c-9cf6-4861-9f2f-3977d379b029.pdf]

Supplementary File 1: Task fMRI Contrasts

The contrasts tests for each task are presented below. Note that the second column (even cope numbers) is the negative of the first column (odd cope numbers). The contrasts with bold style and marked with a star are presented in the paper.

Balloon Analog Risk Task (BART)

Cope	Contrast	Cope	Contrast
**1***	**Accept**	2	neg_Accept
**3***	**AcceptRT**	4	neg_AcceptRT
**5***	**AcceptParametric**	6	neg_AcceptParametric
7	Control	8	neg_Control
**9***	**Explode**	10	neg_Explode
11	ExplodeParametric	12	neg_ExplodeParametric
**13***	**Reject**	14	neg_Reject
15	RejectRT	16	neg_RejectRT
17	RejectParametric	18	neg_RejectParametric
19	AcceptParam-ExplodeParam	20	ExplodeParam-AcceptParam
21	AcceptParam-RejectParam	22	RejectParam-AcceptParam
23	Explode-Reject	24	Reject-Explode

Paired-Associate Memory Task Retrieval (PAMRET)

Cope	Contrast	Cope	Contrast
**1***	**All**	2	neg_All
**3***	**All_RT**	4	neg_All_RT
5	Correct	6	neg_Correct
7	Incorrect	8	neg_Incorrect
9	TruePos	10	neg_TruePos
11	TrueNeg	12	neg_TrueNeg
13	FalsePos	14	neg_FalsePos
15	FalseNeg	16	neg_FalseNeg
17	TruePos_RT	18	neg_TruePos_RT
19	TrueNeg_RT	20	neg_TrueNeg_RT
21	FalsePos_RT	22	neg_FalsePos_RT
23	FalseNeg_RT	24	neg_FalseNeg_RT
25	Control	26	neg_Control
**27***	**TruePos-TrueNeg**	28	TrueNeg-TruePos

29	FalsePos-FalseNeg	30	FalseNeg-FalsePos
**31***	**TruePos-FalsePos**	32	FalsePos-TruePos
33	TrueNeg-FalseNeg	34	FalseNeg-TrueNeg
35	Corr-Incorr	36	Incorr-Corr
37	Correct-Control	38	Control-Correct
39	Incorrect-Control	40	Control-Incorrect
41	TruePos-Control	42	Control-TruePos
43	TrueNeg-Control	44	Control-TrueNeg
45	FalsePos-Control	46	Control-FalsePos
47	FalseNeg-Control	48	Control-FalseNeg
49	All-Control	50	Control-All

Spatial Capacity Task (SCAP)

Cope	Contrast	Cope	Contrast
**1***	**All**	2	neg_All
**3***	**All_rt**	4	neg_All_rt
5	Load1	6	neg_Load1
7	Load3	8	neg_Load3
9	Load5	10	neg_Load5
11	Load7	12	neg_Load7
13	Delay1.5	14	neg_Delay1.5
15	Delay3	16	neg_Delay3
17	Delay4.5	18	neg_Delay4.5
**19***	**LinearUp_load**	20	neg_LinearUp_load
**21***	**LinearUp_delay**	22	neg_LinearUp_delay
23	Load3-load1	24	load1-Load3
25	Load5-load1	26	load1-Load5
27	Load7-load1	28	load1-Load7
29	Load5-load3	30	load3-Load5
31	Load7-load3	32	load3-Load7
33	Load7-load5	34	load5-Load7
35	Delay4_5-delay1_5	36	delay1_5-Delay4_5
37	Delay3-delay1_5	38	delay1_5-Delay3
39	Delay4_5-delay3	40	delay3-Delay4_5

Stop Signal Task

Cope	Contrast	Cope	Contrast
**1***	**Go**	2	neg_Go
**3***	**GoRT**	4	neg_GoRT
5	StopSuccess	6	neg_StopSuccess
7	StopUnsuccess	8	neg_StopUnsuccess
9	StopUnsuccessRT	10	neg_StopUnsuccessRT
**11***	**Go-StopSuccess**	12	StopSuccess-Go
13	Go-StopUnsuccess	14	StopUnsuccess-Go
**15***	**StopSuccess-StopUnsuccess**	16	StopUnsuccess-StopSuccess

Task Switching Task

**1***	**ALL**	2	neg_ALL
**3***	**ALL_rt**	4	neg_ALL_rt
5	CONGRUENT_SWITCH_SHORT	6	neg_CONGRUENT_SWITCH_SHORT
7	CONGRUENT_SWITCH_SHORT_rt	8	neg_CONGRUENT_SWITCH_SHORT_rt
9	CONGRUENT_SWITCH_LONG	10	neg_CONGRUENT_SWITCH_LONG
11	CONGRUENT_SWITCH_LONG_rt	12	neg_CONGRUENT_SWITCH_LONG_rt
13	CONGRUENT_NOSWITCH_SHORT	14	neg_CONGRUENT_NOSWITCH_SHORT
15	CONGRUENT_NOSWITCH_SHORT_rt	16	neg_CONGRUENT_NOSWITCH_SHORT_rt
17	CONGRUENT_NOSWITCH_LONG	18	neg_CONGRUENT_NOSWITCH_LONG
19	CONGRUENT_NOSWITCH_LONG_rt	20	neg_CONGRUENT_NOSWITCH_LONG_rt
21	INCONGRUENT_SWITCH_SHORT	22	neg_INCONGRUENT_SWITCH_SHORT
23	INCONGRUENT_SWITCH_SHORT_rt	24	neg_INCONGRUENT_SWITCH_SHORT_rt
25	INCONGRUENT_SWITCH_LONG	26	neg_INCONGRUENT_SWITCH_LONG
27	INCONGRUENT_SWITCH_LONG_rt	28	neg_INCONGRUENT_SWITCH_LONG_rt
29	INCONGRUENT_NOSWITCH_SHORT	30	neg_INCONGRUENT_NOSWITCH_SHORT
31	INCONGRUENT_NOSWITCH_SHORT_rt	32	neg_INCONGRUENT_NOSWITCH_SHORT_rt
33	INCONGRUENT_NOSWITCH_LONG	34	neg_INCONGRUENT_NOSWITCH_LONG
35	INCONGRUENT_NOSWITCH_LONG_rt	36	neg_INCONGRUENT_NOSWITCH_LONG_rt
**37***	**CONGRUENT-INCONGRUENT**	38	INCONGRUENT-CONGRUENT
**39***	**SWITCH-NOSWITCH**	40	NOSWITCH-SWITCH
41	SWITCH-NOSWITCH_SHORT	42	NOSWITCH_SHORT-SWITCH
43	SWITCH-NOSWITCH_LONG	44	NOSWITCH_LONG-SWITCH
45	CONGRUENT-INCONGRUENT_SHORT	46	INCONGRUENT_SHORT-CONGRUENT
47	CONGRUENT-INCONGRUENT_LONG	48	INCONGRUENT_LONG-CONGRUENT
