# Supplementary material for: Preprocessed Consortium for Neuropsychiatric Phenomics dataset
Source: F1000Res. 2017 Sep 22;6:1262. Originally published 2017 Jul 28. [Version 2] doi: 10.12688/f1000research.11964.2 (PMC5664981; doi:10.12688/f1000research.11964.2)
Supplement: Peer review history

# Peer review: Referee response for version 2

**DOI:** 10.5256/f1000research.13763.r26274

**Published:** 2017-10-31

**Authors:** Angela R. Laird

**Affiliations:** 1 Department of Physics, Florida International University, Miami, FL, USA

The authors have sufficiently addressed my previous concerns, and I now am happy to endorse publication of this manuscript.

I have read this submission. I believe that I have an appropriate level of expertise to confirm that it is of an acceptable scientific standard.

# Peer review: Referee response for version 2

**DOI:** 10.5256/f1000research.13763.r26275

**Published:** 2017-10-18

**Authors:** Anderson M. Winkler

**Affiliations:** 1 Hospital Israelita Albert Einstein, São Paulo, SP, Brazil; 2 Department of Psychiatry, Yale University School of Medicine, New Haven, CT, USA

All suggestions I had for the first version have been addressed in this revised version, and I have no further concerns. I would like to thank and congratulate the authors for making this resource available.

I have read this submission. I believe that I have an appropriate level of expertise to confirm that it is of an acceptable scientific standard.

# Peer review: Referee response for version 1

**DOI:** 10.5256/f1000research.12934.r24602

**Published:** 2017-08-22

**Authors:** Anderson M. Winkler

**Affiliations:** 1 Hospital Israelita Albert Einstein, São Paulo, SP, Brazil; 2 Department of Psychiatry, Yale University School of Medicine, New Haven, CT, USA

First of all, I would like to congratulate the authors for making the dataset available, which should allow interested scientists to explore the data and enrich the research they conduct with more information that can eventually lead to helpful new discoveries. It is remarkable that more than one processing stream was used (FSL and AFNI for functional, and volumetric and surface-based for structural), and further, three different inference approaches were considered, all of which are a great bonus in terms of comparisons among methods.

 I have very few concerns about the current version of the manuscript (v1, dated 28/July/2017):

- Page 2, 1st column, 2nd paragraph of the Introduction: "giving researchers the freedom to fit many different models that incorporate different denoising schemes": as stated, it may suggest that it would be adequate to simply run multiple models, without attention to excess error due to multiple testing. Perhaps a different wording such as "giving researchers the freedom to choose their own denoising schemes" could still accommodate what the authors may have wished to state.
- I cannot find information about the subjects. Who were them? Where was the data collected? With what scanner and sequences? Who approved the protocol? Presumably this information is on reference #1 but it cannot hurt to have that information here.
- It would be good if a few more details on what exactly FMRIPREP does could be given, without having to rely completely on external links that may no longer be available in the future. Even more important considering that results did change with after minor version changes.
- Page 2, 1st column, 4th paragraph of the Methods: As written, a reader may think that the input images to FreeSurfer were those non-linearly aligned to the MNI space, which surely was not the case. But if that was, then the FS analysis would have to re-done as the warps affect thickness and area measurements.
- Page 3, 2nd column: Regarding the masks, one would have thought that using the mask from FEAT/FLAME could have been a good shortcut instead of creating a new one for randomise. Why wasn't that done?
- Page 3, Validation section: The strategy using the mask contours to investigate between-subjects registration is surely not a good one for not showing how the overlap between structures. A hyperslab across subjects would have been more informative. Moreover, the contours shown in Figure 1 are a bit concerning for suggesting somewhat suboptimal registration.
- Still in the Validation section: what exactly is being validated here? It doesn't seem to show that the dataset would be valid or not valid in any particular aspect. Consider investigating some specific validation parameters over different aspects (e.g., registration, bias correction, surface reconstruction, the tasks eliciting expected response, etc), or remove this section altogether, as it can be misleading for suggesting that the dataset is "valid" somehow.
- Page 6: The description of the files is extremely helpful. I note that one of the files listed has extension .h5. Is this HDF5? If yes, please state so. I believe this format was used for the lack of another option, but in fact, this is a great format that probably should in the future be an option for most imaging data we use (both surface-based and volume-based).
- Of the FreeSurfer surfaces, the white is the most important one, not pial or midthickness. The pial is computed after the white already exists, and its exactness depends on the white. The midthickness does not match any particular tissue border, and if one measures surface area from it, that area will depend on thickness, which would make it a poor phenotype. It would be great if the white surface files could be provided.
- Page 7: I find it concerning that information and resources about this dataset are scattered over the internet: There is the current paper (PDF) and its Supplementary Material on F1000, then there are results stored in NeuroVault, source code on Github and Zenodo, and finally, a Docker container on DockerHub. Could not a copy of all these pieces be on a single place that can be simply downloaded and maintained on the long term, e.g., in DataDryad? How can the readers be sure that all these links will be alive in 10 or 20 years?

I have read this submission. I believe that I have an appropriate level of expertise to confirm that it is of an acceptable scientific standard, however I have significant reservations, as outlined above.

# Response

**Published:** 2017-09-16

**Authors:** Krzysztof J. Gorgolewski

**Affiliations:** Center for Reproducible Neuroscience, Stanford University, USA

Dr. Winkler,

Thank you for the detailed review. Your comments helped us to improve the manuscript in the following way:

- We have applied the suggested changes to the second paragraph of the introduction
- We have added information about the subjects
- We have clarified that the FS analysis was done in parallel with the alignment.
- We have added a few more details about preprocessing and an overview figure. Furthermore we have mode sure that the online documentation of the version of FMRIPREP used to generate this data has been deposited in the Internet Archive for long term preservation.
- We have clarified why different mask strategies were applied.
- We have added the hyperslab figure to the manuscript.
- The 95% and 85% overlap brain mask overlap contours show good agreement across the normalized masks with signal dropout in areas usually affected by susceptibility distortion artifacts. The 100% overlap is much worse since it requires voxels to be present in all of the 1,969 evaluated masks.
- We have changed the header ‘Validation’ to ‘Selected Results’ to not give the wrong impression that we validated the analyses.
- We added clarification on the file format for the .h5 files (indeed it’s HDF5!)
- The white surface is provided (both in GIfTI and native FreeSurfer formats) - we made this information more prominent.
- We have clarified that there is long-term storage of the data and code.

# Peer review: Referee response for version 1

**DOI:** 10.5256/f1000research.12934.r24599

**Published:** 2017-08-08

**Authors:** Angela R. Laird

**Affiliations:** 1 Department of Physics, Florida International University, Miami, FL, USA

This Data Note reports on the availability of an fMRI dataset from the Consortium for Neuropsychiatric Phenomics (CNP), which includes both original and processed data. The publication of shared fMRI datasets is strongly encouraged to amplify our community's efforts in promoting open science. Although dataset publications are on the rise, unfortunately, only a handful currently exist. I am delighted to see this work being published, and expect that it may serve as a representative fMRI dataset publication in the future. With that in mind, I think it would be helpful to revise the manuscript to include a more detailed description of the data, acquisition methods, and individual tasks. 

 Introduction: The first paragraph of the Introduction is extremely brief and should be expanded to include a description of the purpose of the study, participants, and the experimental protocol (imaging and behavioral). Only a very short mention of these three important aspects of the study are provided and collapsed into a single (somewhat awkward) first sentence. Such brevity might limit a reader's understanding of the overall context of the data that are being shared. The second sentence of the Introduction alludes to "relationships" being "answered" - this should be restated and expanded to more fully describe what questions may be asked from these data - again, this relates to the overall purpose of the CNP project. While such additional descriptions will result in a longer paper, the information will be helpful in allowing readers to understand how their specific research questions may be addressed by a deeper exploration of these data. Lastly, the Introduction should also state where the data may be downloaded and a summary of the different unprocessed and processed files that have been shared.

 Methods: Demographic information on the participants should be provided, as well as a statement of IRB approval. A description of the MRI scanner should be included, along with the data acquisition parameters. Each of the fMRI tasks should be fully described - this will help clarify subsequent reference to different conditions (e.g., “accept, explode, reject”). Much of the Methods is written for those who are already very familiar with the software packages that are utilized. It would be helpful to improve accessibility by including a brief description of some of the newer, less ubiquitious software tools. In particular, given how the Methods is framed around use of FMRIPREP, a short intro should be included. The total numbers of participants for each of the tasks (shown on page 3) doesn’t agree with the numbers of task datasets discarded for being incomplete - please note why the additional participants were omitted from the final dataset. Overall, the flow of the Methods section could be improved by adding a workflow or pipeline figure that summarizes the different analysis steps and the versions of the data (e.g., volume vs. surface approaches). 

 I’m not convinced that “Dataset validation” is an appropriate heading. Figure 1 is a good sanity check, but it’s not clear how Figures 2 - 6 are a validation.

 Data and software availability: Some readers may not be familiar with the BIDS format - please add a description of this. In addition, some readers may be looking for information about the DICOM and NIfTI images, so an explicit mention may be helpful.

 Minor comments:

 - page 2: first report of DVARS is capitalized, but later mentions are not

 - page 2: "Temporal derivatives were added to all task regressors to compensate ***for*** variability in the haemodynamic response function" 

 - page 3: typo - “unsuccesful”

 - ensure past tense used consistently throughout Methods (e.g., “tasks data ***were*** discarded”, “no correct answers ***were*** registered” on page 3).

I have read this submission. I believe that I have an appropriate level of expertise to confirm that it is of an acceptable scientific standard, however I have significant reservations, as outlined above.

# Response

**Published:** 2017-09-16

**Authors:** Krzysztof J. Gorgolewski

**Affiliations:** Center for Reproducible Neuroscience, Stanford University, USA

Dr. Laird,

Thank you for your review and comments. They were very helpful in preparation of a new revision of the paper.

- We have extended the introduction to give a clearer view on the purpose and possibilities this dataset gives. In the methods sections, we have given demographic information on the participants, as well as IRB approval and a description of the MRI scanner, scanning parameters and tasks.
- We have changed the heading ‘Dataset validation’ to ‘Selected results’.
- We have expanded the description of preprocessing and introduction to the FMRIPREP package.
- We have clarified that not all subjects performed all of the tasks.
- We added a data processing overview figure.
- We have spelled out the BIDS acronym and added a reference to a paper with more information.
- We have added information about NIFTI and GIFTI file formats (DICOM files are not part of this dataset).
- Furthermore, in accordance with the review, we have fixed the reported typos and changed the tense of the paper.
